# Structural and functional analyses of human DDX41 DEAD domain

**DOI:** 10.1007/s13238-016-0351-9

**Published:** 2016-12-07

**Authors:** Yan Jiang, Yanping Zhu, Weicheng Qiu, Yong-Jun Liu, Genhong Cheng, Zhi-Jie Liu, Songying Ouyang

**Affiliations:** 1 National Laboratory of Biomacromolecules, Institute of Biophysics, Chinese Academy of Sciences, Beijing, 100101 China; 2 Baylor Research Institute, Baylor Scott and White Health, Dallas, TX 75246 USA; 3 Department of Microbiology, Immunology and Molecular Genetics, University of California Los Angeles, Los Angeles, CA 90095 USA; 4 Institute of Molecular and Clinical Medicine, Kunming Medical University, Kunming, 650500 China

**Dear Editor,**

DEAD-box proteins, which are named after the strictly conserved amino acid sequence Asp-Glu-Ala-Asp, were first identified as a distinct family in the late 1980s when alignments based on eight homologues of the yeast eIF4A highlighted the presence of several conserved motifs (Linder et al., 1989). DEAD-box proteins are widely distributed in different life forms, ranging from bacteria to human and constitute the largest RNA helicase family (Jiang et al., 2016). They are involved in many aspects of RNA metabolism, such as splicing, mRNA export, transcriptional and translational regulation, ribosome biogenesis and RNA decay (Rocak and Linder, 2004). The core of DEAD-box proteins is organized into two major domains. Domain 1 (DEAD domain) consists of motifs Q, I (Walker A, P-loop), II (Walker B, DEAD-box), Ia, GG, Ib and III, whereas domain 2 (Helicase domain) consists of motifs IV, V and VI. Different motifs are involved in nucleotide binding (Q, I and II), RNA binding (Ia, Ib, IV and V) and ATP hydrolysis (III and possibly VI). Compared with the two conserved domains, the N- and C-terminal regions are variable and divergent. Their functions are not fully characterized, but they are thought to confer their own specificity on different proteins (Hogbom et al., 2007).

The recognition of pathogen-associated molecular patterns (PAMPs) of pathogens by pattern recognition receptors (PRRs) is important for the induction of type I interferons (IFN) (Medzhitov and Janeway, 2000). DDX41, a member of the DEAD-box proteins, containing a disordered N-terminal region, a DEAD domain and a Helicase domain (Fig. 1A), was identified as an intracellular DNA sensor in myeloid dendritic cells (mDCs) by Yong-Jun Liu’s group. They showed that DDX41 directly binds DNA and STING via its DEAD domain and triggers activation of signaling mediated by mitogen-activated protein kinases TBK1 and transcription factor IRF3, resulting IFN production (Zhang et al., 2011). DDX41 can also detect bacterial secondary messengers like cyclic di-GMP (c-di-GMP) and cyclic di-AMP (c-di-AMP), leading to formation of a complex with STING. This complex transmits the signal of bacterial intrusion to TBK1-IRF3 and activates the interferon response (Parvatiyar et al., 2012). Phosphorylation of Tyr414 of DDX41 is a pre-requisite for foreign dsDNA recognition and recruitment of STING. Besides, BTK’s kinase domain can bind the DEAD domain of DDX41 (Lee et al., 2015). After immune response, DDX41 will be ubiquitinated by TRIM21 through K48-mediated linkage for degradation. The ubiquitination sites are Lys9 and Lys115 (Zhang et al., 2013). Somatic DDX41 mutations have been reported in the study of sporadic acute myeloid leukemia (AML) syndrome (Ding et al., 2012). A familial MDS/AML syndrome characterized by long latency and germline mutations in DDX41 gene is also identified (Polprasert et al., 2015). DDX41 can associate with spliceosomal proteins, and its defects lead to loss of tumor suppressor function due to altered pre-mRNA splicing and RNA processing (Polprasert et al., 2015). Although DDX41 plays important roles in innate immunity and diseases, the precise mechanism as well as the extent of involvement the protein in these processes is poorly understood.

**Figure 1:**
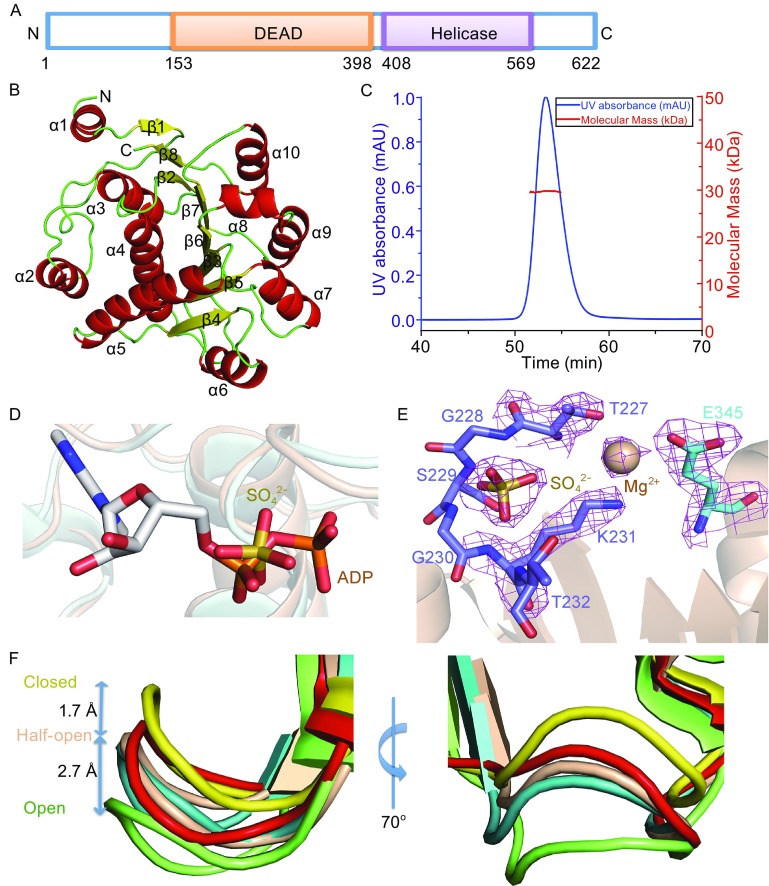
**Overall structure of hDDX41 DEAD domain**. (A) Domain organization of hDDX41, composing of DEAD domain and Helicase domain from N to C terminus. (B) A ribbon representation of DEAD domain with secondary structural elements labeled. Helix, sheet and loop are colored in red, yellow and green, respectively. (C) The multiangle static light scattering result of DEAD domain protein. The calculated molecular weight is 29.3 kDa. (D) Structure alignment of hDDX41 (wheat) to the structure of DDX5 (light blue) in complex with ADP (in white sticks from PDB code: 3FE2) reveals that the bound SO_4_^2−^ (yellow sticks) is located in approximately the same position of α-phosphate of ADP. The bound SO_4_^2−^ of hDDX41 and ADP are shown sticks. (E) The SO_4_^2−^ is coordinated by P-loop and the Mg^2+^ is coordinated by T227, K231 from P-loop and E345 from motif II. The electron density map (2Fo-Fc) of SO_4_^2−^, Mg^2+^, T227, K231 and E345 is contoured at 1.0 σ. (F) Superposition of the P-loop of hDDX41 with SO_4_^2−^ (wheat), Prp28 (yellow), DDX3X with AMP (red), DDX5 with ADP (cyan), VASA with ANP (green)

Here, we report the crystal structure of human DDX41 (hDDX41) DEAD domain complexed with an SO_4_^2−^ and an Mg^2+^ to 2.26 Å resolution. There are strong interactions between different motifs to stabilize the whole structure. The P-loop presents in a half-open conformation. The DEAD domain protein can bind ADP and AMP but not ATP *in vitro* because of the steric hindrance. Most mutated amino acids related with familial MDS/AML are conserved. In addition, the N-terminal disordered region (amino acid 1–152) is shown targeting hDDX41 protein to the nucleus. Our study provides basic structural information for further researches on hDDX41 biological function and valuable insights for the treatment of DDX41-related diseases in the future.

The crystal structure of hDDX41 DEAD domain was solved by molecular replacement using the structure of DDX5 domain I (PDB code: 3FE2) as the search model and refined to 2.26 Å resolution with an R factor of 0.19 (R_free_ = 0.23). Details of data collection and refinement statistics are listed in Table S1. The crystal used for data collection belonged to space group *P*2_1_. One asymmetric unit consists of two molecules of the protein based on the calculated solvent content of 44.15%. The hDDX41 DEAD domain consists of an α/β fold, which is similar to those observed for other members of the DEAD-box proteins for which structures are available. The overall structure consists of ten α-helices (α1–α10) and a β-sheet formed by eight β-strands (β1–β8). Helices α1–α5 are located on one side of the β-sheet, while helices α6–α10 are located on the other side (Fig. 1B). Although there are two monomers in one asymmetric unit, PDBePISA (http://www.ebi.ac.uk/msd-srv/prot_int/cgi-bin/piserver) predicted a monomeric biological assembly. In agreement with this prediction, results of the static light scattering analysis indicated that the DEAD domain exists as a monomer in solution (Fig. 1C).

Although ADP or AMP was added in molar excess to the protein prep before crystallization, no electron density was observed for these ligands. Instead, clear electron density for one SO_4_^2−^ probably originating from the crystallization solution, was observed in the nucleotide-binding site. When aligned to the structure of DDX5 in complex with ADP (PDB code: 3FE2), the SO_4_^2−^ overlaps with the position of α-phosphate of ADP (Fig. 1D). The P-loop is seen forming the SO_4_^2−^ binding pocket and an Mg^2+^ is observed coordinated by T227, K231 from P-loop and E345 from motif II (Fig. 1E). Mutating P-loop or motif II to alanine resulted in insoluble protein. The structure of DDX41 DEAD domain solved by us contains motif Q, P-loop, motif II, motif Ia, motif Ib and motif III. These structural elements are located at either β-strand-loop or helix-loop transitions (Fig. S1). There are many interactions between the different motifs, which probably stabilizes the overall structure. These interactions are listed in Table S2.

The P-loop is responsible for the nucleotide binding. Fig. 1F shows superposition of the P-loop of hDDX41 with SO_4_^2−^ (wheat), Prp28 (yellow, PDB code 4NHO), DDX3X with AMP (red, PDB code 2I4I), DDX5 with ADP (cyan, PDB code 3FE2), VASA with ANP (green, PDB code 2DB3). It seems that the P-loop is not restricted to one open or closed conformation but has a flexibility that allows it to adapt to different conformations depending on the binding ligands. The most closed conformation is found in Prp28, which leaves no room for any ligand. The most open conformation is found in VASA, which has enough space for ANP. The P-loop of DDX3X with AMP and DDX5 with ADP adopt the same half-open conformation compared with Prp28 and VASA. Although there is no nucleotide in the solved hDDX41 structure, the SO_4_^2−^ bound P-loop adopts a half-open conformation. The P-loop has a shift in Cα-atom positions by up to 2.7 Å between the open and half-open states, and by up to 1.7 Å between the half-open and closed states (Fig. 1F). The conformation of the P-loop seems to be determined by the nucleotide phosphates, and longer phosphate tails result in a more open loop. During our manuscript submission, Omura et al. reported two similar crystal structures of the DDX41 DEAD domain with root-mean-square deviation (RMSD) of 0.5 Å between the main chain Cα atoms of the 330 amino acids (Fig. S2) (Omura et al., 2016).

The binding affinity of hDDX41 DEAD domain with ATP, ADP, AMP, c-di-GMP and cGAMP was detected *in vitro* by Thermal Shift Assay (TSA) and Isothermal Titration Calorimetry (ITC) (Fig. 2A). The thermal denaturation profiles indicated a T_m_ of 41°C for unliganded hDDX41 DEAD domain. Addition of ATP, c-di-GMP, and cGAMP to the protein did not increase the T_m_. However, the Tm increased in presence of ADP and AMP by 2.2°C and 3.9°C, respectively, implying that the protein probably binds ADP and AMP but not ATP. There was no detectable interaction between hDDX41 DEAD domain with c-di-GMP and cGAMP, although the full length protein is reported to bind c-di-GMP (Parvatiyar et al., 2012). ITC results suggested a binding affinity of 31 µM and 61 µM for ADP and AMP, respectively. Furthermore, the ITC results indicated that the DEAD domain of DDX41 does not interact with ATP, c-di-GMP, or cGAMP, which is consistent with the TSA results. AMP or ADP could be modeled into the binding pocket of hDDX41 DEAD domain by superimposing the crystal structure of VASA (PDB code: 2DB3) and DDX5 (PDB code: 3FE2) over that of DDX41. The adenosine moiety fits well into the pocket and the α-, β-phosphate can also be accommodated. However, a γ-phosphate as in the case of ATP would clash with T227 of the P-loop (Fig. 2B). The negatively charged binding pocket is not big enough for ATP (Fig. 2B). This may explain why we could not detect any significant affinity of the protein for ATP *in vitro.*

**Figure 2:**
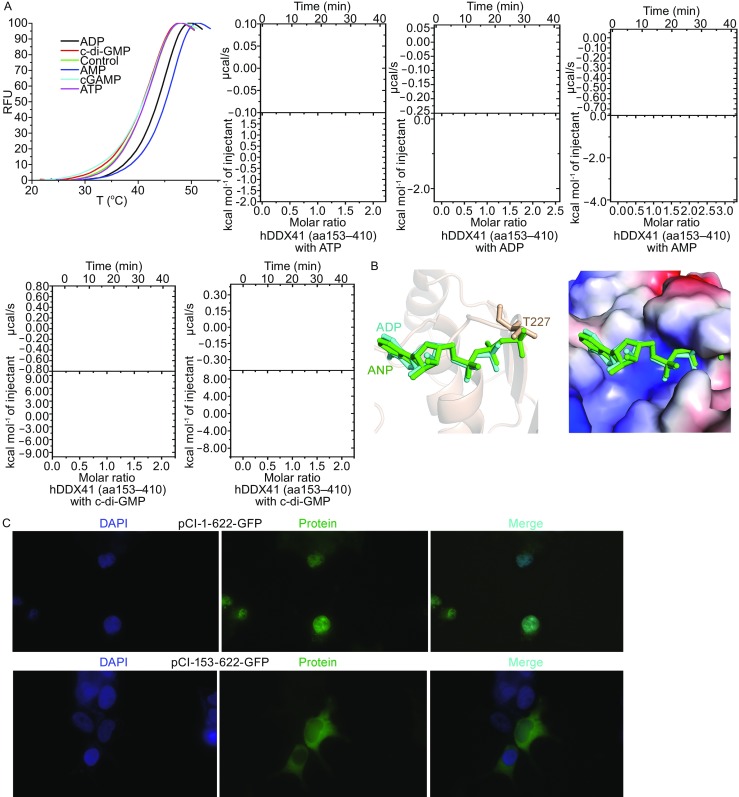
**The binding of hDDX41 DEAD domain with different molecules and N-terminal region targets hDDX41 to the nucleus**. (A) Thermal Shift Assay and Isothermal Titration Calorimetry of hDDX41 DEAD domain protein with ATP, ADP, AMP, c-di-GMP and cGAMP. (B) Left: the modeled ADP and ANP are colored in cyan and green. The γ-phosphate of ANP clashes with T227 of hDDX41. Right: surface electrostatic potential representation of the nucleotide binding pocket. Blue, positive potential; red, negative potential. The positively charged binding pocket is not big enough for ANP binding. (C) Fluorescence microscopy of HEK293T cells transfected with expression plasmids for GFP-tagged hDDX41 full length protein (1–622) and GFP-tagged hDDX41 N-terminal region deleted truncation (153–622). Nuclei are stained with DAPI

hDDX41 is frequently mutated in familial MDS/AML (Polprasert et al., 2015). We analyzed the conservation of hDDX41 amino acids using the ConSurf Server (http://consurf.tau.ac.il/) and found out that the mutated amino acids associated with MDS/AML are conserved (Fig. S3). Of the nine mutations identified, seven mutations (p.M155I, p.R164W, p.F183I, p.A225D, p.E247K, p.P321L, p.I396T) are locate in the DEAD domain, suggesting hDDX41 function is more sensitive to mutations in DEAD domain than Helicase domain. MDS/AML is now the only reported disease related to hDDX41 protein. However, the relationship between the mutations and disease is still unknown. hDDX41 could serve as a drug target and our study provides a structural basis for disease treatment.

Secondary structure prediction of hDDX41 (Fig. S4) reveals that the N-terminal region (aa 1–160) is disordered. In addition, the role of this region is unclear. Interestingly, the N-terminal 1–194 amino acids of a homologue of DDX41, Abstrkt, from *Drosophila* play a role in the translocation of the protein to the nucleus (Abdul-Ghani et al., 2005). Alignment of the N-terminal regions of the two proteins shows that they share 41.9% identity (Fig. S5). We generated GFP-fusions of full length hDDX41 and truncations of hDDX41 missing aa 153–622 and monitored their cellular localization after transfection in HEK293T cells. GFP-fusions containing the full length protein showed nuclear localization while deletion of aa 153–622 resulted in distinct punctate cytoplasmic distribution and loss of nuclear localization (Fig. 2C). Taken together, we conclude that the N-terminal disordered region (aa 1–152) of hDDX41 can target the protein to the nucleus.

DDX41 is reported to bind dsDNA and c-di-GMP directy by DEAD domain. However, the affinity of DEAD domain with dsDNA and c-di-GMP can’t be detected *in vitro.* Different lengths of dsDNA and c-di-GMP were tested by TSA, ITC and Surface Plasmon Resonance (SPR) assay, but no binding was detected. As intracellular condition is much more complicated, DDX41’s functions in innate immunity may need other components or depends on both domains. Besides c-di-GMP, STING is also a direct sensor of c-di-GMP (Burdette et al., 2011). We solved the complex structure of hSTING (aa 139–379) and c-di-GMP (Ouyang et al., 2012). One c-di-GMP binds to the interface of STING dimer with a unique mode. We mixed c-di-GMP with hDDX41 DEAD domain before crystallization, but there is no electron density for c-di-GMP in the structure.

In summary, we report the crystal structure of hDDX41 DEAD domain complexed with an SO_4_^2−^ and an Mg^2+^ to 2.26 Å resolution. But the mechanism of hDDX41 recognition with foreign dsDNA and c-di-GMP remains unclear. Our study provides basic structural information for further researches on DDX41 biological functions and the treatment of DDX41-related diseases in the future.

## Electronic supplementary material

Below is the link to the electronic supplementary material.

Supplementary material 1 (PDF 500 kb)
