# Erratum to: Structural and functional analyses of human DDX41 DEAD domain

**DOI:** 10.1007/s13238-016-0361-7

**Published:** 2017-01-13

**Authors:** Yan Jiang, Yanping Zhu, Weicheng Qiu, Yong-Jun Liu, Genhong Cheng, Zhi-Jie Liu, Songying Ouyang

**Affiliations:** 1 National Laboratory of Biomacromolecules, Institute of Biophysics, Chinese Academy of Sciences, Beijing, 100101 China; 2 Baylor Research Institute, Baylor Scott and White Health, Dallas, TX 75246 USA; 3 Department of Microbiology, Immunology and Molecular Genetics, University of California Los Angeles, Los Angeles, CA 90095 USA; 4 Institute of Molecular and Clinical Medicine, Kunming Medical University, Kunming, 650500 China

## Erratum to: Protein Cell 2016 DOI 10.1007/s13238-016-0351-9

In the original publication of this article Fig. 2 has been incorrectly published. The correct Fig. 2 is provided in this erratum.

**Figure 2:**
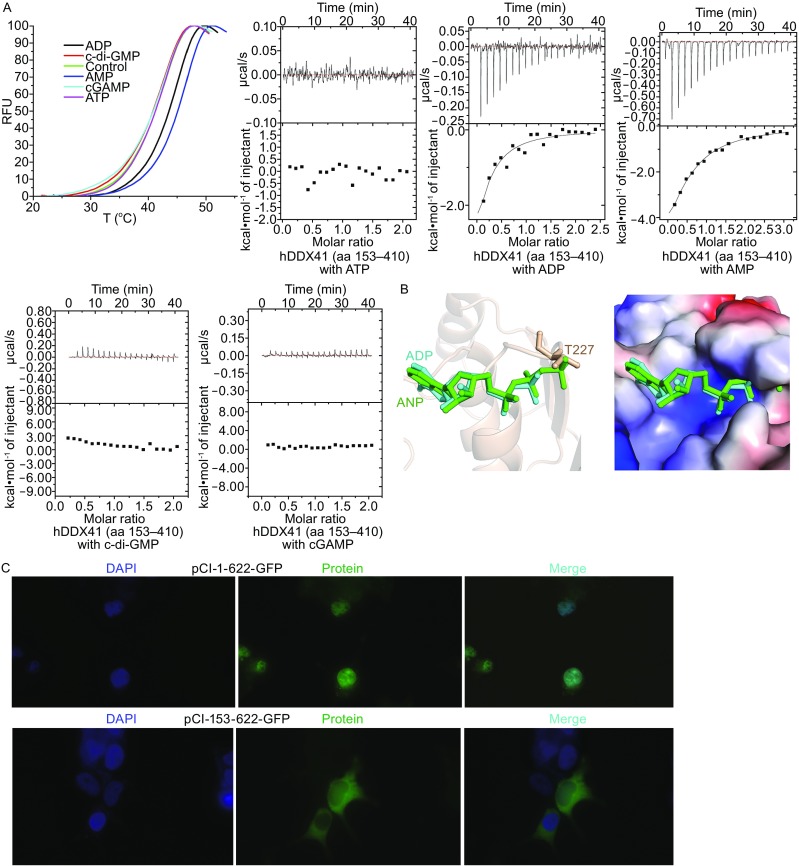
The binding of hDDX41 DEAD domain with different molecules and N-terminal region targets hDDX41 to the nucleus. (A) Thermal Shift Assay and Isothermal Titration Calorimetry of hDDX41 DEAD domain protein with ATP, ADP, AMP, c-di-GMP and cGAMP. (B) Left: the modeled ADP and ANP are colored in cyan and green. The γ-phosphate of ANP clashes with T227 of hDDX41. Right: surface electrostatic potential representation of the nucleotide binding pocket. Blue, positive potential; red, negative potential. The positively charged binding pocket is not big enough for ANP binding. (C) Fluorescence microscopy of HEK293T cells transfected with expression plasmids for GFP-tagged hDDX41 full length protein (1–622) and GFP-tagged hDDX41 N-terminal region deleted truncation (153–622). Nuclei are stained with DAPI

